# Cutaneous Small-Vessel Vasculitis Induced by Escitalopram: A Case-Based Brief Review of the Literature

**DOI:** 10.7759/cureus.62776

**Published:** 2024-06-20

**Authors:** Achilleas Betsikos, Maria Mousia, Evangelia Simopoulou, Evanthia Gazouni, Stauroula Magaliou, Eleni Paschou, Nikolaos Sabanis

**Affiliations:** 1 1st/2nd Department of Internal Medicine, General Hospital of Trikala, Trikala, GRC; 2 Department of Pathology, General Hospital of Trikala, Trikala, GRC; 3 1st Department of Internal Medicine, General Hospital of Trikala, Trikala, GRC; 4 Department of Nephrology, General Hospital of Trikala, Trikala, GRC; 5 Department of General Practice & Family Medicine, 10th Local Medical Unit of Giannouli, Larisa, GRC

**Keywords:** cutaneous small-vessel vasculitis, selective serotonin reuptake inhibitor (ssri), purpura, escitalopram, leukocytoclastic vasculitis (lcv)

## Abstract

A 65-year-old male with multiple comorbidities and recently diagnosed with diabetic kidney disease developed upper and lower extremity rash following escitalopram initiation for his depressive mood. Clinical assessment and skin biopsy confirmed cutaneous small-vessel vasculitis (CSVV), prompting drug discontinuation and oral methylprednisolone therapy. The resolution of the rash was achieved within a week. This rare case of CSVV induced by escitalopram highlights the importance of timely recognition and management of drug-induced CSVV and adds to the limited literature on selective serotonin reuptake inhibitor-associated CSVV.

## Introduction

Selective serotonin reuptake inhibitors (SSRIs) are the cornerstone of pharmacotherapy for major depressive disorder and a variety of mood disorders. Since their introduction, SSRIs have revolutionized the treatment of these psychiatric conditions due to their efficacy and relatively tolerable side effect profile compared to earlier antidepressants. SSRIs work by selectively inhibiting the reuptake of serotonin into presynaptic neurons, thereby increasing the availability of serotonin in the synaptic cleft and enhancing serotoninergic neurotransmission [1].

Despite their widespread use and general safety, SSRIs are associated with a range of adverse effects that warrant caution. Common side effects include gastrointestinal disturbances, sexual dysfunction, and weight gain, which can impact patient adherence to therapy [2]. Furthermore, SSRIs have been linked to dermatological reactions, such as rash and urticaria, and even rarer conditions, such as cutaneous small-vessel vasculitis (CSVV), toxic epidermal necrolysis, and Stevens-Johnson syndrome [3]. Here, we report a clinical case of CSVV induced by escitalopram administration.

## Case presentation

A 65-year-old man presented at the nephrology outpatient department of the hospital complaining about a rash on both upper and lower extremities that developed two days ago. Ten days before the presentation, the patient consulted a psychiatrist due to his depressed mood, following the diagnosis of diabetic kidney disease he had received six months earlier. They agreed on cognitive behavioral therapy in combination with escitalopram pharmacotherapy. Other notable medical history included coronary artery bypass graft surgery following an episode of acute myocardial infarction 20 years ago, as well as coronary angioplasty seven years ago, type II diabetes mellitus, hypertension, heart failure with mid-range ejection fraction, peripheral artery disease, benign prostatic hyperplasia, hyperuricemia, and non-alcoholic fatty liver disease. His medications included rosuvastatin, furosemide, valsartan, carvedilol, amlodipine, empagliflozin, sitagliptin, insulin glargine, febuxostat, aspirin, omeprazole, and alprazolam.

**Figure 1:**
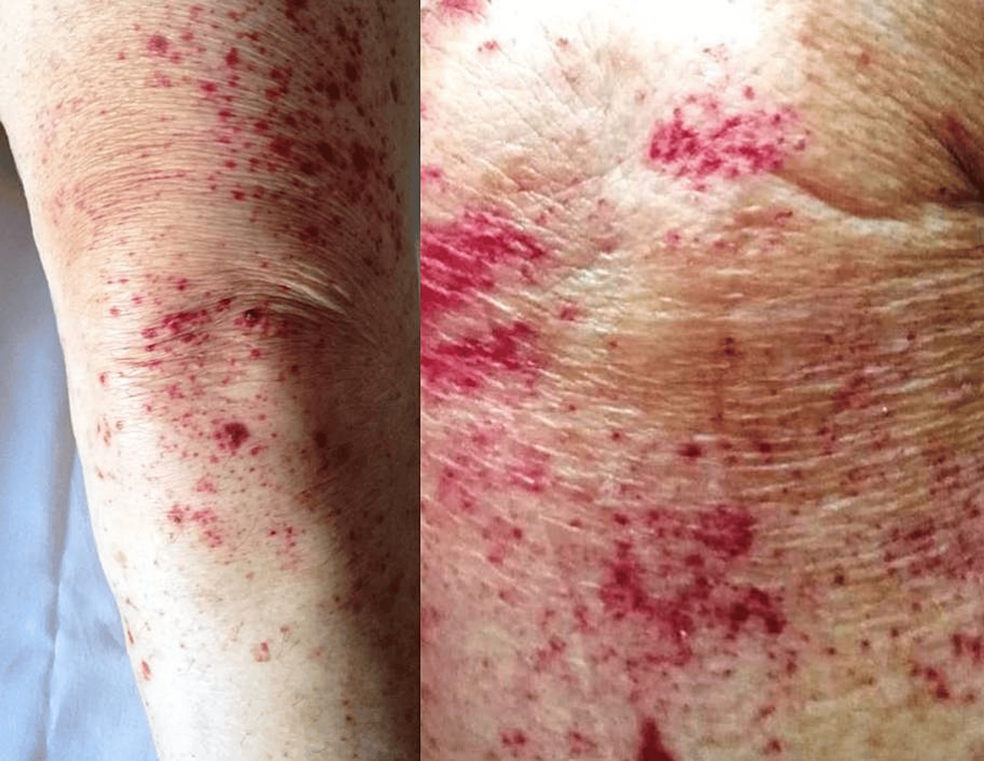
Small-vessel cutaneous vasculitis: confluent petechiae rash presented as clusters of small, slightly elevated red spots typical of palpable purpura on both upper and lower extremities of a 65-year-old man after selective serotonin reuptake inhibitor administration.

On presentation, the patient was anxious and concerned about the rash (Figure 1). There was neither pruritus nor pain but clusters of small, slightly elevated red spots typical of palpable purpura. A dermatologist was consulted and a complete panel of laboratory tests and a skin biopsy were ordered. The rest of a detailed physical examination was unremarkable. CSVV was considered to be our working diagnosis. Escitalopram was withheld and a short, tapered course of oral methylprednisolone was administered for 10 days. The patient’s anxiety was quelled by explaining the benign nature of this condition.

**Figure 2:**
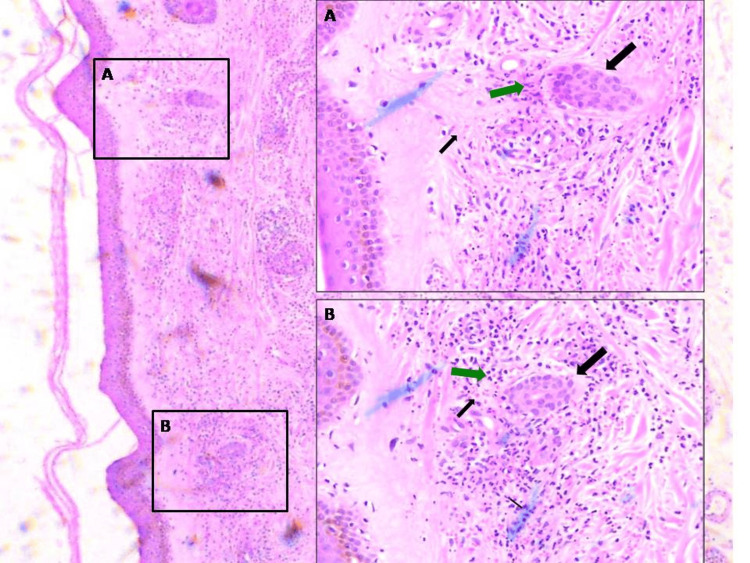
A, B: Cutaneous small-vessel vasculitis: histopathological findings of skin biopsy stained by hematoxylin-eosin (×100, ×400) showing infiltration with polymorphonuclear neutrophils in and around the vessel walls, endothelial edema, and fibrinoid necrosis (big black arrow); abundant nuclear debris (leukocytoclasia) (green arrow); and extravasated erythrocytes (thin black arrow).

In the follow-up one week after the initial examination, the lesions had completely resolved. Furthermore, escitalopram was replaced by fluoxetine according to the suggestions of the patient’s psychiatrist. The results of the skin biopsy revealed infiltration with polymorphonuclear neutrophils in and around the vessel walls accompanied by signs of activation and death of neutrophils illustrated by abundant nuclear debris (leukocytoclasia); evidence of tissue damage was noted with the presence of endothelial edema, extravasated erythrocytes, and coexistence of eosinophilic and lymphocytic infiltrates, as well as fibrinoid necrosis (Figure 2). Laboratory and imaging results including immunological tests, inflammation markers, virological and bacteriological testing, repeated microscopic examination of the urine, and computed tomography evaluation were not noteworthy. More results are shown in Table 1. Therefore, by excluding other possible causes of systemic disease, we established this skin-isolated small-vessel vasculitis induced by escitalopram as the final diagnosis.

**Table 1:** Laboratory findings. WBC = white blood cells; INR = international normalization ratio; aPTT = activated partial thromboplastin time; ESR = erythrocyte sedimentation rate; SGOT = serum glutamic-oxaloacetic transaminase; SGPT = serum glutamic pyruvic transaminase; LDH = lactate acid dehydrogenase; CPK = creatine phosphokinase; PSA = prostate-specific antigen; CEA = carcinoembryonic antigen; AFP = alpha fetoprotein; HBsAg = hepatitis B surface antigen; anti-HCV = antibodies to hepatitis C virus; HIV = human immunodeficiency virus; ASTO = antistreptolysin O titer; ANCA = anti-neutrophil cytoplasm antibodies

Parameters	Patient’s result	Normal range
WBC	6.860	4–10.8 × 10^3^/μL
Hematocrit	33.1	37.7–47.9%
Hemoglobin	11.2	11.8–17.8 g/dL
Platelets	260	150–350 × 10^3^/μL
INR	1.21	
aPTT	34.8	24–35 seconds
ESR	26	mm/hour
Glucose	126	75–115 mg/dL
Urea	67	10–50 mg/dL
Creatinine	2.45	0.40–1.10 mg/dL
Sodium	143.5	136–143 mg/dL
Potassium	4.76	3.5–5.1 mg/dL
SGOT	17	5–40 IU/L
SGPT	160	10–37 IU/L
LDH	220	135–225 IU/L
CPK	121	24–190 IU/L
C-reactive protein	0.86	<0.7 mg/dL
PSA	1.386	<4 ng/mL
CEA	2.84	<5 ng/mL
AFP	0.48	0.74–7.29 U/mL
CA-19.9	19.99	<37 U/mL
HBsAg	0.21	<1 S/CO
Anti-HCV	0.13	<1 pg/mL
HIV I, II	0.19	<1 S/CO
ASTO	36	<200 U/mL
Complement C3	1.15	0.85–1.80 g/L
Complement C4	0.22	0.10–0.40 g/L
Antinuclear antibodies	12.4	<40 AU/mL
Anti-DNA-ds	2.16	<30 IU/mL
ANCA-C	Negative	<1/20
ANCA-P	Negative	<1/20
Cryoglobulins	Negative	
Serum protein electrophoresis	Normal	
Plasma and urine immunofixation	Normal	
Urine microscopic examination	No active sediment	
Urine protein	510	0.02–0.150 g/d

## Discussion

Vasculitides encompass a heterogeneous group of disorders characterized by the inflammation of blood vessels. These conditions can affect vessels of all sizes and can involve multiple organ systems, manifesting with a variety of clinical presentations ranging from mild skin lesions to life-threatening illnesses. Etiologically, vasculitides may be idiopathic, infectious, or associated with systemic diseases such as an autoimmune disease, hematological disorder, or even malignancy. Pathogenesis often involves immune complex deposition, autoantibodies, and aberrant immune response, resulting in vessel wall inflammation. Diagnostic approaches include serological testing, imaging, and histopathological examination of the affected tissues. Management strategies are tailor-made to the specific type of vasculitis, typically involving immunosuppressive therapy to mitigate inflammation and prevent target organ damage [4,5].

CSVV is a specific type of vascular inflammation limited to the capillaries, venules, and arterioles of the skin. It is usually a diagnosis of exclusion (Table 2). It is of paramount clinical importance that other systemic causes are ruled out. In its drug-induced type, patients develop lesions within 7-21 days after treatment initiation. It is considered to be a benign condition, which mostly resolves spontaneously after a single episode. Nevertheless, severe cases may warrant systemic corticosteroid administration. Histopathologically, CSVV is characterized by leukocytoclastic vasculitis, where neutrophil and eosinophil infiltrates are evident within and around the vessel wall; fibrinoid necrosis is also present inside or within the vessel walls. There may be evidence of endothelial damage such as endothelial swelling, sloughing and necrosis, extravasated red blood cells, and abundant perivascular nuclear dust due to neutrophil activation and cell death. Immunofluorescence studies, if needed, typically reveal perivascular deposition of immunoglobulins and complement components [4,6]. In the majority of CSVV cases, the above characteristic lesions are located around the small venules of the upper dermis while the presence of tissue eosinophilia is usually correlated with a drug-induced etiology, as observed in our case [7].

**Table 2:** Causes of cutaneous small-vessel vasculitis.

Idiopathic - primary	
Drugs	Non-steroidal anti-inflammatory drugs
	Beta-lactam antibiotics
	Sulfonamides
	Vancomycin
	Allopurinol
	Amiodarone
	Thiazide diuretics (furosemide)
	D-penicillamine
	Tumor necrosis factor-alpha inhibitors
	Propylthiouracil
	Phenytoin
	Sodium valproate
	Oral anticoagulants such as warfarin
	Selective serotonin reuptake inhibitors
Infections (bacterial, viral, parasitic)	Group A *Streptococcus* infections
	Staphylococcus
	Gonococcus
	*Mycobacterium tuberculosis*, *Mycobacterium chelonae*, *Mycobacterium leprae*
	Meningococcus
	Pseudomonas
	Hepatitis B, hepatitis C
	Human immunodeficiency virus
	Cytomegalovirus
	Parvovirus B19
	SARS-CoV-2
	Influenza A virus
	Flavivirus (dengue fever)
	Plasmodium
	Loa-loa filarial disease
	Toxocara canis
Autoimmune diseases	Henoch-Shonlein purpura
	IgA vasculitis
	Systemic lupus erythematosus
	Rheumatoid arthritis
	Sjogren’s syndrome
	Cryoglobulinemia
	Behçet’s disease
	Anti-neutrophil cytoplasm antibody-positive vasculitis
Malignancies	Solid tumors
	Leukemia
	Lymphoma
	Myeloproliferative/Myelodysplastic disorders
Miscellaneous	Ulcerative colitis
	Crohn’s disease

Clinically, patients present with palpable purpura predominantly on the lower extremities, although constitutional symptoms, including fever, arthralgia, and malaise, may be present. A thorough history and physical examination, supplemented by laboratory tests and skin biopsy, are essential for accurate diagnosis. Management of CSVV focuses on addressing the underlying cause when identifiable. In our case, withdrawal of the offending drug in conjunction with methylprednisolone led to the rapid resolution of the rash.

To our knowledge, only a few cases of SSRI-induced CSVV have been reported. By scrutinizing the available literature, we have found a subset of cases similar to ours but none involving escitalopram. In the family of SSRIs, sertraline [8] and paroxetine [9] have been linked to CSVV while fluoxetine has been reported to induce another form of vasculitis, urticarial vasculitis [10]. Moreover, in the spectrum of antidepressant drugs, maprotiline [11], a tricyclic antidepressant, sibutramine [12], a serotonin-norepinephrine reuptake inhibitor, and vortioxetin, a serotonin modulator and stimulator, were involved in cases of CSVV. Regardless of the causative medication, this rare case underscores the importance of recognizing this adverse drug reaction so that the clinician proceeds to its prompt discontinuation [13].

## Conclusions

This case highlights the rare occurrence of CSVV induced by escitalopram, adding to the scant literature on SSRI-induced CSVV. Although it is generally considered benign and self-resolving, some cases may necessitate systemic corticosteroid therapy. Prompt recognition and discontinuation of the offending drug are crucial in managing this adverse drug reaction. Of note, as almost every type of vasculitis may involve the skin, even as their first target organ manifestation, it is crucial to go the long way of exclusion before establishing the diagnosis of CSVV. Finally, clinicians should remain vigilant for such clinical presentations, ensuring individualized management to optimize patient outcomes.
